# Increased Small Dense LDL and Decreased Paraoxonase Enzyme Activity Reveals Formation of an Atherogenic Risk in Streptozotocin-Induced Diabetic Guinea Pigs

**DOI:** 10.1155/2013/860190

**Published:** 2013-03-27

**Authors:** Mutay Aslan, Filiz Ozcan, Ertan Kucuksayan

**Affiliations:** Department of Medical Biochemistry, Akdeniz University, Faculty of Medicine, 07070 Antalya, Turkey

## Abstract

This study aimed to investigate LDL subfraction distribution as well as serum cholesteryl ester transfer protein (CETP), lecithin-cholesterol acyltransferase (LCAT), and paraoxonase (PON1) activity in streptozotocin-induced diabetic guinea pigs. *Materials/Methods*. Guinea pigs were given a single intraperitoneal (ip) injection of streptozotocin (STZ) and animals having fasting blood glucose levels greater than 200 mg/dl, were considered diabetic. Protein levels of LCAT and CETP were determined via ELISA. Paraoxonase activity was measured kinetically by the formation of phenol while LDL subfraction analysis was done by disc polyacrylamide gel electrophoresis. *Results*. Plasma glucose and high-density lipoprotein (HDL) cholesterol were significantly increased while total cholesterol and LDL cholesterol were significantly decreased in diabetic guinea pigs compared to controls. LDL subfraction analysis revealed a significant decrease in nonatherogenic LDL-2 subfraction and a significant increase in atherogenic LDL-4 subfraction in diabetic guinea pigs compared to controls. Plasma CETP and PON1 levels were significantly decreased while LCAT showed no significant difference in diabetic guinea pigs compared to controls. *Conclusion*. Decreased non-atherogenic LDL-1, LDL-2 subfractions, increased small dense LDL-4 subfraction, and decreased PON1 activity, reveals formation of an atherogenic risk in diabetic guinea pigs. Decrease in CETP levels supports the observed increase in HDL cholesterol levels in diabetic guinea pigs.

## 1. Introduction

Type 1 diabetic patients, even those who are normolipidemic, present increased risk of premature atherosclerosis. This suggests that normal values in lipid profile can mask alterations in the composition and distribution of the denser LDL subclasses, whose characteristics make them potentially more atherogenic [1]. The association between LDL cholesterol levels and ischemic heart disease (IHD) is well established; however, it has been shown that 35% of patients with a cholesterol level less than 200 mg/dl develop IHD [2].

Circulating LDL particles are heterogeneous with respect to size, density, composition, and physicochemical properties [3]. Using gradient gel electrophoretic analysis of isolated LDL, two distinct LDL phenotypes have been documented [4]. Pattern B shows a predominance of small, dense LDL particles, while pattern A reveals a higher proportion of large, more buoyant LDL particles. Irrespective of the approach used to characterize LDL particles and of the case definition, dense LDL particles were more prevalent among IHD case patients than among IHD-free control subjects [1].

The respective contributions of the dense LDL phenotype and of lipoprotein-lipid levels to the subsequent development of IHD were examined in a subsample of men involved in the prospective phase of the Que'bec Cardiovascular Study [5]. Obtained results showed that the presence of small, dense LDL particles may be associated with an increased risk of subsequently developing IHD in men. Results also suggested that the risk attributed to small LDL particles may be partly independent of the concomitant variation in plasma lipoprotein-lipid concentrations.

In accordance with these observations, adults with type 1 diabetes have been reported to have comparable to or better lipoprotein profile than nondiabetic adults [6]. Yet, people with type 1 diabetes suffer macrovascular complications and death at earlier ages than nondiabetics [7]. The possible mechanisms behind macrovascular complications in type 1 diabetes can be explored in detail in the presence of an appropriate animal model. Guinea pigs have been reported to be a suitable animal model to study lipoprotein metabolism due to the fact that they carry the majority of the cholesterol in LDL [8]. Guinea pigs also possess cholesterol ester transfer protein and lipoprotein lipase activities, which results in reverse cholesterol transport and metabolic cascades equivalent to the human situation [8]. In this context, we aimed to investigate LDL subfraction distribution as well as serum CETP and LCAT in streptozotocin-induced diabetic guinea pigs. Paraoxonase activity was also measured in diabetic animals to determine the possible contribution of altered enzyme activity in vascular complications associated with the disease.

## 2. Materials and Methods

### 2.1. Animals

Male guinea pigs weighing 200–250 g were obtained from Akdeniz University Experimental Animal Laboratory. Animals were housed at 22 ± 28°C with 12 h light/dark cycles and 50 ± 5% humidity and provided with standard laboratory chow ad libitum. All animal procedures were reviewed and approved by the animal ethics committee at Akdeniz University. Diabetes was induced by intraperitoneal (ip) injection of streptozotocin (STZ, Alexis Biochemicals, USA). Streptozotocin freshly dissolved in 0.1 M cold citrate buffer (pH 4.5) was injected at a single dose of 280 mg/kg body weight following overnight fasting as previously described [9]. This dose of STZ induced diabetes in guinea pigs as previously reported [10]. However, a guinea pig is more resistant to drug-induced diabetes than a rat [11] and our success rate was 20% in these animals. Fasting blood glucose levels were measured 3 days after STZ injection via a glucometer (Accu-Check Go, Roche Co.), and guinea pigs having blood glucose levels greater than 200 mg/dl were included in the study. 

### 2.2. Serum Lipid Concentration Measurements

Guinea pigs were anesthetized intraperitoneally with a mixture of ketamine (25 mg/kg, Richter Pharma AG, Wels, Austria) and xylazine hydrochloride (8 mg/kg, Alfasan International B.V., Woerden, Holland). Blood samples were collected by cardiac puncture under anesthesia. Serum was harvested after centrifugation at 1,500 xg at room temperature for 10 min. Total cholesterol (TC), HDL-cholesterol, and triacylglycerol (TG) were measured on Roche Cobas 8000 Modular Analyser (Basel, Switzerland) via enzymatic colorimetric methods.

### 2.3. LDL Subfraction Analysis

LDL subfraction analysis was performed electrophoretically by use of high-resolution 3% poylacrylamide gel tubes and the Lipoprint LDL System (Quantimetrix, Redondo Beach, CA, USA) according to the manufacturer's instructions [12]. Briefly, 25 *μ*L of sample was mixed with 200 *μ*L of liquid loading gel. The loading gel contained Sudan Black B dye to stain the lipoproteins. The resulting mixture was added to the top of precast 3% polyacrylamide gel tubes. After photopolymerization at room temperature for 35 min, samples were electrophoresed for 1 h 5 min (3 mA/gel tube). Densitometry was performed by Microtek ArtixScan M1 system and data was analyzed by Quantimetrix software (Lipoware-Research version) as previously described [12, 13]. In this method, VLDL remains in the origin (retention factor (Rf) = 0.0), whereas HDL migrates at the front (Rf = 1.0). In between, several bands can be detected: MID bands C, B, and A, which correspond mainly to intermediate-density lipoprotein (IDL), as well as the LDL bands. LDL1 and LDL2 bands correspond to large, buoyant LDL particles, whereas bands LDL3 and above correspond to sdLDL particles. VLDL and the proportion (%) of the cholesterol mass (mg/dl) of LDL subfractions over the total LDL-cholesterol mass were calculated by Quantimetrix software.

### 2.4. CETP and LCAT Measurement

Serum CETP concentrations were analyzed by a commercial ELISA test kit (47-CETHU-E01; ALPCO, Salem, NH, USA) according to the manufacturer's instructions. Test wells were coated with anti-CETP Monoclonal Ab. CETP in the sample was captured by the antibody in the 1st incubation. After the 1st incubation and washing to remove all of the unbound material, horseradish-peroxidase-(HRP-) labeled anti-CETP Monoclonal Ab was added. After the 2nd incubation and subsequent washing, substrate solution was added. Next, stop reagent was added and the intensity of color that develops was measured at 492 nm by a microplate reader. A standard curve of absorbance values of known CETP standards was plotted as a function of the logarithm of CETP standard concentrations (*μ*g/mL) using the GraphPad Prism Software program for windows version 5.03, (GraphPad Software Inc.). CETP concentrations in the samples were calculated from their corresponding absorbance values via the standard curve.

Serum LCAT concentrations were analyzed by a commercial ELISA test kit (47-LCAHU-E01;ALPCO, Salem, NH, USA) according to the manufacturer's instructions. Test wells were coated with anti-LCAT monoclonal antibody, which binds with LCAT in the sample. After the first incubation and washing to remove all of the unbound material, HRP-labeled anti-LCAT was added. After the second incubation and subsequent washes, the antibody/LCAT/enzyme complex was incubated with a substrate solution and terminated with a stop reagent. The intensity of color that develops was measured at 492 nm by a microplate reader. A standard curve of absorbance values of known LCAT standards was plotted as a function of the logarithm of LCAT standard concentrations (*μ*g/mL) using the GraphPad Prism Software program for windows version 5.03, (GraphPad Software Inc.). LCAT concentrations in the samples were calculated from their corresponding absorbance values via the standard curve.

### 2.5. PON1 Activity Measurement

Serum PON1 activity was determined by a commercial assay kit (ZMC catalogue number 0801199; Zeptometrix Corporation, Buffalo, NY, USA) according to the manufacturer's instructions. This assay is based on the principle that PON1 catalyzes the cleavage of phenyl acetate, resulting in phenol. The rate of formation of phenol was measured by monitoring the increase in absorbance at 270 nm, at 25°C. One unit of arylesterase activity is equal to 1 *μ*M of phenol formed per minute. The activity is expressed in kU/L, based on the extinction coefficient of phenol of 1310 M^−1^cm^−1^ at 270 nm at pH 8.0 and at 25°C. Blank samples containing water were used to correct for nonenzymatic hydrolysis.

### 2.6. Statistical Analysis

Data were analyzed using Sigma Stat (version 2.03) statistical software for Windows, and a *P*  value < 0.05 was considered statistically significant.

## 3. Results

### 3.1. Blood Glucose and Lipid Profile

There was a significant increase in serum glucose of diabetic guinea pigs compared to controls (Table 1). In contrast, total cholesterol and LDL cholesterol was significantly decreased in the diabetic group as compared to control (Table 1). Although not significant, a pronounced decrease was also observed in TG levels of diabetic animals compared to controls. There was a significant increase in HDL cholesterol of diabetic guinea pigs in comparison with control (Table 1).

### 3.2. Changes in LDL Subfraction Pattern between Control and Diabetic Guinea Pigs

**Figure 1:**
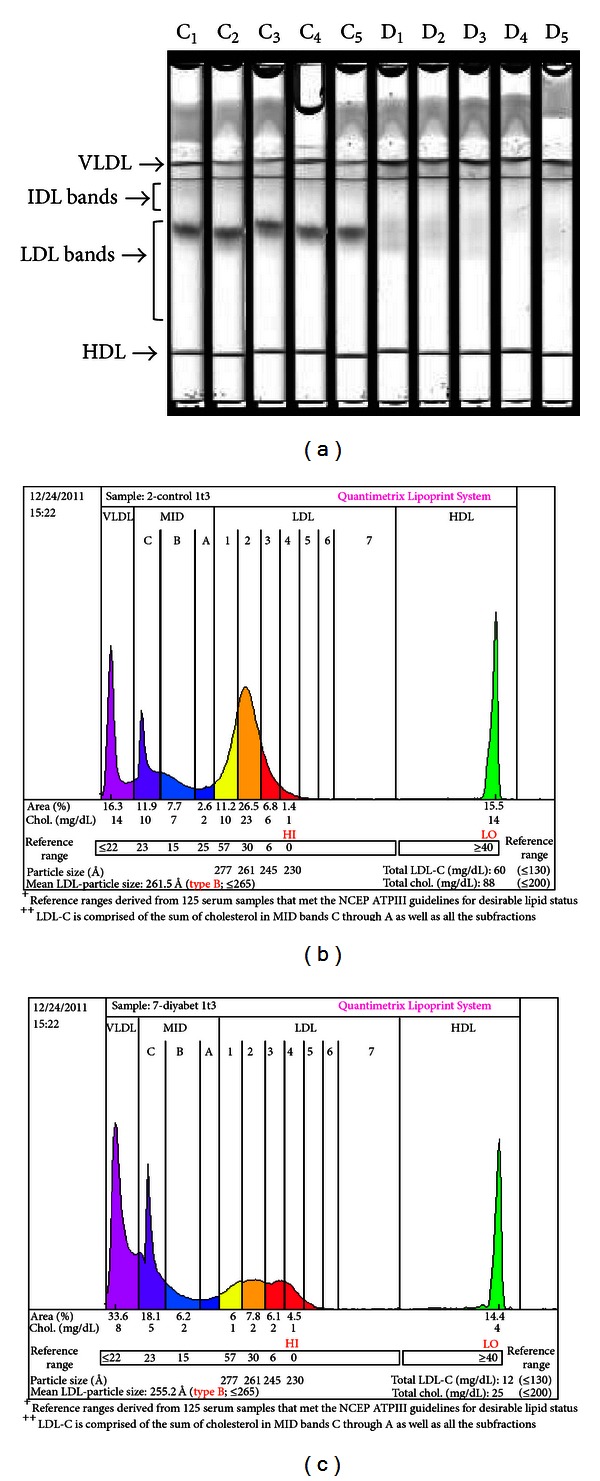
Electrophoretic separation of lipoproteins on the Quantimetrix Lipoprint LDL System. (a) Image of electrophoretic migration of 10 gel tubes. C: control; D: diabetes. (b) Densitometric scan of a control sample, gel tube C1. (c) Densitometric scan of a diabetic sample, gel tube D1.

Figure 1(a) shows 10 gel tubes (5 controls and 5 diabetic) after completion of electrophoresis. Electrophoretic migration was from the top of the tube to the bottom. Separation was achieved via particle size based on the sieving action of the gel. Chylomicrons remained in the loading gel; VLDL was the slowest migrating band while HDL migrated to a further distance. The LDL particles were separated in the middle part of the gel. Bands corresponding to large, buoyant LDL particles showed clear decrease in intensity in the diabetic group. Figures 1(b) and 1(c) are densitometric scans of tube C1 and tube D1. A significant increase in IDL-C and small dense LDL-4 fraction (Figures 1(b) and 1(c) and Table 2) and a significant decrease in large buoyant LDL-2 particles (Figures 1(b) and 1(c) and Table 2) were seen in the diabetic group compared to control.

### 3.3. Serum CETP, LCAT, and PON1 Activity

**Figure 2:**
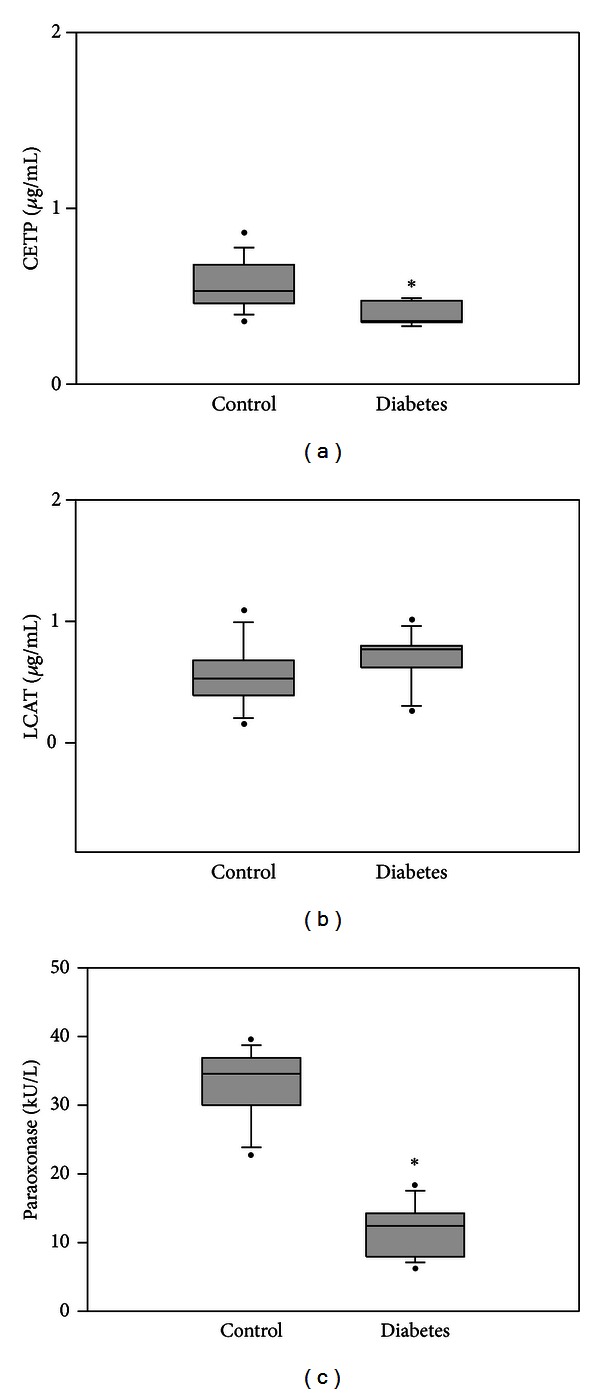
(a) Box plot graph data of plasma cholesteryl ester transfer protein (CETP) measured in control (*n* = 15) and diabetic (*n* = 5) guinea pigs. Statistical analysis was by Student's *t*-test, *P = 0.037. (b) Box plot graph data of lecithin-cholesterol acyltransferase (LCAT) measured in control (*n* = 15) and diabetic (*n* = 5) guinea pigs. (c) Paraoxonase (PON1) activity measured in control (*n* = 15) and diabetic (*n* = 5) guinea pigs. Statistical analysis was by Student's *t*-test, *P < 0.001.

Box plot graph data of CETP and LCAT protein content in healthy and diabetic guinea pigs are shown in Figures 2(a) and 2(b), respectively. The boundary of the box closest to zero indicates the 25th percentile, the line within the box marks the median, and the boundary of the box farthest from zero indicates the 75th percentile. Whiskers above and below the box indicate the 90th and 10th percentiles. CETP protein (mean ± SD) measured in the diabetic group (0.4 ± 0.07 *μ*g/mL) was significantly lower compared to control (0.57 ± 0.16 *μ*g/mL). No significant difference was observed in LCAT protein between control and diabetic groups with measured levels of 0.56 ± 0.26 and 0.70 ± 0.24 *μ*g/mL, respectively. A significant decrease was observed in serum PON1 activity (mean ± SD) in the diabetic group (11.66 ± 4.22 kU/L) compared to control (32.88 ± 5.50 kU/L).

## 4. Discussion

To our knowledge this is the first study evaluating both lipid profile and LDL subfractions in diabetic guinea pigs. Streptozotocin-induced pathophysiological changes under hyperglycemic conditions in animals are very similar to human diabetes [14]. Streptozotocin is taken up by pancreatic beta-cells via the glucose transporter GLUT2, resulting in DNA fragmentation by alkylation of DNA bases, thus leading to beta-cell necrosis [14]. Streptozotocin injected into guinea pigs herein induced a severe diabetic state in 20% of the animals characterized by marked loss of body weight, hyperglycemia, and marked polyuria, a diabetic state closely resembling T1DM.

The suitability of guinea pigs to study changes on cholesterol and lipoprotein metabolism was shown in previous studies [8]. One of the most relevant similarities between guinea pigs and humans is that the majority of circulating cholesterol is transported in LDL [15]. Major key points that support the use of guinea pigs as models for human cholesterol and lipoprotein metabolism include the presence of plasma CETP activity [16] which is a critical component for human reverse cholesterol transport [17]. Guinea pigs also have LCAT and lipoprotein lipase (LPL) activities that are important in the remodeling of plasma lipoproteins, which result in the formation of lipoprotein subclasses [16].

Although lipid levels in patients with T1DM have been found to be comparable to or better than nondiabetic adults, adults with T1DM still are known to be at higher risk for atherosclerotic disease compared with the general population. Fasting lipid profiles were determined in 652 type 1 diabetic patients and 764 nondiabetic control subjects as part of the ongoing prospective Coronary Artery Calcification in Type 1 Diabetes (CACTI) study [6]. This study reported lower TC, LDL-cholesterol, and TG and higher HDL-cholesterol levels in both men and women type 1 diabetic patients compared to controls [6]. We have observed similar findings of lower TC, LDL-cholesterol, and TG and higher HDL-cholesterol in our study conducted on guinea pigs. The levels of serum TC, TG, VLDL-, HDL-, and LDL-cholesterol in guinea pigs reported herein are in accordance with previous studies performed on guinea pigs [18, 19].

In agreement with previous studies conducted on humans [20, 21] we have similarly observed an increase in small dense LDL and IDL in diabetic guinea pigs compared to controls. Plasma lipoprotein subfractions were studied in 9 type 1 diabetic patients during conventional insulin therapy and in 14 healthy controls. The cholesterol concentrations of IDL and the minor component of LDL was found to be significantly higher in diabetic patients than in control subjects [20]. Similar findings were also reported in a separate study which investigated LDL subclasses in 12 normolipidemic type 1 diabetic patients and 11 healthy controls. Plasma concentration of denser LDL subfractions was higher in type 1 diabetic patients versus control subjects while the LDL profile was skewed toward the lighter subclasses in the healthy controls [21].

Plasma CETP and LCAT are two major enzymes that are involved in the remodeling of plasma lipoproteins. Cholesteryl ester transfer protein facilitates the transfer of cholesteryl esters from HDL to apoB100-containing lipoproteins in exchange for TG [22]. Deficiency in CETP leads to large HDL particles and increased apoA-1 [23]. In line with previous studies conducted on humans [24] we have observed significantly decreased plasma CETP levels in diabetic guinea pigs compared to non-diabetic controls. Decreased CETP levels observed in diabetic guinea pigs may thus lead to significantly increased HDL cholesterol levels observed in the diabetic group. 

Lecithin cholesterol acyltransferase catalyzes the esterification of cholesterol, especially in HDL, by promoting transfer of fatty acids from lecithin to cholesterol [25]. We observed no significant difference in serum LCAT levels between diabetic and control guinea pigs. Our observation confirms previous studies on humans which report no significant change in LCAT levels in type 1 diabetes [26, 27].

The presence of serum PON1 activity has been reported in guinea pigs [28], but to our knowledge this is the first study evaluating PON1 activity in diabetic guinea pigs. As stated previously, HDL-associated PON1 retards oxidation of LDL [29] and therefore inhibits the progression of atherosclerosis [30]. As stated previously, HDL-associated PON1 retards oxidation of LDL [29] and therefore inhibits the progression of atherosclerosis [30]. The reduction of accumulating oxidized lipids in LDL reduces monocyte activation and production of interleukin-8 and monocyte colony-stimulating factors which induce adhesion of monocytes to endothelial surface [31]. PON1 also contributes to the attenuation of atherosclerosis development by leading to the formation of lysophosphatidylcholine, which, in turn, stimulates HDL binding and HDL-mediated macrophage cholesterol efflux via the ABCA1 transporter [32].

In line with previous studies conducted on humans [33, 34], we have similarly observed decreased serum PON1 activity in diabetic guinea pigs. Increased oxidative stress and altered antioxidant-protective mechanisms reported in type 1 diabetic animal models [35, 36] can lead to reduced PON1 due to inhibition of the enzyme by its substrates, lipid peroxides [37]. This may provide a mechanism by which decreased PON1 activity promotes atherosclerosis and increased risk for vascular complications in type 1 diabetes. Moreover, low activity of PON1 in the diabetic group suggests that HDL could show functional deficiency in type 1 diabetic patients, despite high HDL-cholesterol levels [38].

In conclusion, this study conducted on STZ-induced diabetic guinea pigs shows that type 1 diabetes generates a state of increased atherosclerotic risk by promoting an increase in small dense LDL-4 subfraction. Although the decrease in CETP levels supports the observed increase in HDL cholesterol levels in diabetic guinea pigs, low activity of PON1 in the diabetic group suggests that HDL could show functional deficiency. Decreased PON1 activity in diabetic guinea pig also reveal increased susceptibility to vascular complications.

## Figures and Tables

**Table 1:** Mean blood glucose and lipid profile in type 1 diabetic and control guinea pigs.

Parameter	Control (*n* = 15)	Diabetes (*n* = 5)	*P* value
Glucose			
(mg/dL)	139.20 ± 9.49	213.40 ± 13.18*	<0.001
TG			
(mg/dL)	76.13 ± 28.56	39.80 ± 22.03	0.060
(mmol/L)	0.86 ± 0.32	0.45 ± 0.24	
Total cholesterol			
(mg/dL)	53.27 ± 10.41	31.20 ± 5.26*	0.023
(mmol/L)	1.38 ± 0.27	0.81 ± 0.14*	
LDL			
(mg/dL)	34.31 ± 14.87	13.53 ± 2.01*	0.007
(mmol/L)	0.89 ± 0.38	0.35 ± 0.05*	
VLDL			
(mg/dL)	12.00 ± 1.98	10.47 ± 1.83	0.145
(mmol/L)	0.31 ± 0.05	0.27 ± 0.05	
HDL			
(mg/dL)	3.00 ± 1.56	7.6 ± 2.79*	<0.001
(mmol/L)	0.08 ± 0.04	0.20 ± 0.07*	

Values are mean ± SD.

**Table 2:** LDL subfraction analysis in type 1 diabetic and control guinea pigs.

Parameter	Control (*n* = 15)	Diabetes (*n* = 5)	*P* value
IDL-C (%)	15.20 ± 2.04	18.82 ± 1.09*	0.002
IDL-B (%)	5.59 ± 1.84	6.99 ± 1.13	0.128
IDL-A (%)	1.30 ± 1.06	0.95 ± 1.60	0.586
LDL-1 (%)	8.26 ± 3.12	5.39 ± 0.58	0.060
LDL-2 (%)	19.98 ± 4.96	6.10 ± 1.71*	<0.001
LDL-3 (%)	10.67 ± 2.48	5.37 ± 1.4*	<0.001
LDL-4 (%)	1.97 ± 1.56	4.1 ± 0.27*	0.008

Values are mean ± SD.

## Abbreviations

CETP: Cholesteryl ester transfer protein
CVD: Cardiovascular disease
HDL: High-density lipoprotein
IHD: Ischemic heart disease
IP: Intraperitoneal
LCAT: Lecithin-cholesterol acyltransferase
LDL: Low-density lipoprotein
LPL: Lipoprotein lipase
PON1: Paraoxonase
STZ: Streptozotocin
T1DM: Type 1 diabetes mellitus
TC: Total cholesterol
TG: Triacylglycerol.
